# Effect of Propolis on PPP2R1A and Apoptosis in Cancer Cells

**DOI:** 10.1155/bri/5538068

**Published:** 2025-01-15

**Authors:** Burak Durmaz, Latife Merve Oktay Çelebi, Ayşe Çekin, Ayshan Ahadova, Nur Selvi Günel, Hatice Kalkan Yıldırım, Ali Mert Özgönül, Eser Yıldırım Sözmen

**Affiliations:** ^1^Department of Medical Biochemistry, Faculty of Medicine, Near East University, Nicosia, Cyprus; ^2^Department of Medical Biochemistry, Faculty of Medicine, Ege University, İzmir, Türkiye; ^3^Department of Medical Biology, Faculty of Medicine, Ege University, İzmir, Türkiye; ^4^Department of Food Engineering, Faculty of Engineering, Ege University, İzmir, Türkiye; ^5^Department of Medical Biochemistry, Faculty of Medicine, Tınaztepe University, İzmir, Türkiye

**Keywords:** apoptosis, metastasis, PPP2R1A, propolis, tumor suppressor

## Abstract

Recently, it has been shown that protein phosphatase 2A (PP2A) dysfunction was common in many cancer types and was mediated by various inactivation mechanisms. Although many research studies observed antitumor effect of propolis extracts in various types of cancer, the mechanism of effect are still obscure. In this study, we investigated the effect of propolis on PPP2R1A expression and its relationship with apoptosis in the SW-620 (colorectal cancer), DU-145 and PC-3 (prostate cancer), and MCF-7 (breast cancer) cell lines, with WI-38 (healthy fibroblast) cells serving as the control. Moreover, we aimed to investigate the impact of propolis on apoptosis by analyzing apoptosis markers such as tumor necrosis factor-related apoptosis-inducing ligand (TRAIL), APAF-1, and caspases-3, -8, and -9. Propolis samples were extracted, and their phenolic compounds were quantified using LC-MS/MS. The RealTime Cell Analysis System-xCELLigence (RTCA-SP) device and software were employed to assess cell viability and cytotoxicity of the propolis samples. The IC_50_ values for propolis were determined (298 μg/mL for SW-620, 185.6 μg/mL for DU-145, 250.7 μg/mL for PC − 3, 292.9 μg/mL for MCF-7, and 311.2 μg/mL for WI-38). Subsequently, the effects of propolis on PPP2R1A expression and apoptosis markers (TRAIL, Apaf-1, and caspases-3, -8, and -9) were analyzed. When we compared the healthy cell lines to cancer cell lines, a statistically significant increase in caspase-3 (3.62-fold) and in TRAIL (4.38-fold) was observed in the SW-620 cell line after the application of propolis. In addition, in the PC-3 cell line, a 1.4-fold increase in caspase-8 was observed compared with the healthy cell line, which is also statistically significant. Our findings indicated that propolis increased the PPP2R1A levels and apoptosis markers in cancer cell lines. It has been suggested that high PPP2R1A levels induced by propolis treatment might activate the apoptosis pathway. In this study, the inducible effect of propolis on PPP2R1A levels, identified as a new target for cancer treatment, was demonstrated for the first time. The findings suggest that propolis holds promise as a potential cancer therapy by increasing PPP2R1A levels, a key molecule in cancer treatment.

## 1. Introduction

The International Agency for Research on Cancer (IARC) of the World Health Organization (WHO) has stated that the number of new cancer cases is estimated to increase by 22.5% in the next 2 decades [1]. Considering this figure today, the use of cochemotherapeutic products is becoming more important [2]. As a cochemotherapy, propolis is believed to reduce the severity of the side effects of chemotherapy [3]. Propolis is a natural product produced in the hive by honey bees [4] and serves as a natural source of antioxidants and antitumor molecules, which more than 180 compounds (polyphenols, phenolic acid and its esters, phenolic aldehydes, ketones, and flavonoids) were identified in propolis extracts [5–10]. Recent studies have shown that propolis-induced apoptosis pathways in cancer cells and was proposed as potential chemotherapeutic or chemopreventive anticancer drugs. In recent studies, it has been observed that the combination of propolis and chemotherapy reduced the proliferation of cancer cells through by inducing apoptosis, stopping the cell cycle and downregulating the expression of phosphatidylinositol-3 kinase (PI3K) [3]. However, the other mechanisms of the anticarcinogenic effects of propolis have not been clearly determined till now.

Protein phosphorylation reversible by kinases and phosphatases is an important pathway for signal transduction in cancer cells. PP2A is one of the important cellular serine-threonine phosphatases [11]. Recent studies have emphasized that orally available and nontoxic PP2A-reactivating small molecules showed potent anticancer effects both alone and in combination with kinase inhibitors. PP2A is involved in the regulation of various cellular pathways, including the control of cell growth [12]. Next generation cancer drugs aim to suppress different proteins and to activate PP2A [13]. Recently, PP2A dysfunction mediated by various inactivation mechanisms has been noted in many cancer types [14], and it can be assumed that functional PP2A is crucial to prevent tumor development and/or progression.

PP2A, a holoenzyme, exists in two isoforms encoded by different genes. Isoform *α* consists of 590 amino acids and is encoded by the PPP2R1A gene. Isoform *β* consists of 602 amino acids and is encoded by PPP2R1B. PPP2R1A controls DNA replication, signal transduction, cell migration, cell death, and other physiological processes in the body [15]. Therefore, protecting PP2R1A levels as the key target in cancer treatment is the focus of this study.

Notably, in one study, these functionally more disruptive variants were also found in the Catalog of Somatic Mutations in Cancer (COSMIC) or affected at the same amino acid as cancer-associated PPP2R1A variants. Reported functional studies of these cancer-associated A*α* variants have confirmed their increased oncogenic potential in cancer cell growth, migration, and drug resistance, but as this is the first reported case of PPP2R1A with a malignancy, it is unclear whether this is mainly coincidental or part of the clinical phenotype of the more severely affected PPP2R1A subgroup [16]. Therefore, the present study is the first attempt to determine whether PPP2R1A has an effect on cancer using different cancer cell lines and healthy fibroblast cells and propolis, whose phenolic content we determined due to its heterogeneity.

In normal cells, activation of initiating caspases (caspases-2, -8, -9, and -10) results from cell death signaling in either of two ways: (a) extrinsic, death receptor pathway or (b) intrinsic, mitochondrial pathway. The extrinsic pathway is mediated by the stimulation of tumor necrosis factor-associated ligands and tumor necrosis factor-related apoptosis-inducing ligand (TRAIL) R1 and R2 receptors followed by the activation of caspase-8 and then caspase-3 [17]. The intrinsic pathway is regulated by the mitochondria, where the proapoptotic proteins such as cytochrome-c are released into the cytosol and activate the multimeric complex (apoptosome), which is composed of apoptosis protease activating factor-1 (APAF-1), and procaspase-9 leads to caspase-9 activation followed by caspase-3 activation [18]. Although the anticancer effect of propolis has been linked to apoptosis induction in cancer cells, its anticancer effect is not explained by this induction alone.

In this study, we aimed to investigate the possible induced effect of propolis on PPP2R1A in cancer cells in terms of SW-620, DU-145, PC3, MCF-7 cancer cell line, and WI-38 healthy cell line. We focused on the apoptotic pathways in order to explain the mechanism of anticancer effect of propolis.

## 2. Materials and Methods

### 2.1. Preparation of Propolis Samples

Propolis was purchased from a local company called “Ceyan Arıcılık” located in the Ayaş district of Ankara. The samples were obtained from Isparta Şarkikaraağaç region (38° 04′ 45.98″ N/31° 21′ 59.00″) in June. The propolis samples were stored in −20°C in order to facilitate the grinding process with propolis gaining a brittle structure. The particle size was determined as 35 mesh (0.5 mm) by applying the sieve analysis method to the grinded samples. Powdered propolis extracts (1 g) were solved in 100 mL of ethanol + phosphate buffer (v/v: 10/90) solution by incubating in 30°C for 24–72 h [10, 19].

**Figure 1:**
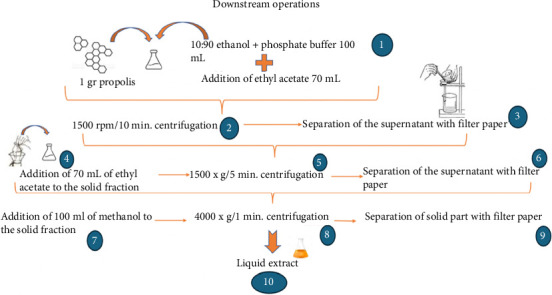
Downstream operations to obtain the propolis sample for study.

Downstream operations are shown in Figure 1.

a. 70 mL of ethyl acetate was added on it and the samples were kept at room temperature for 10 min. After centrifugation at 1500 × g/5 min, the mixture was filtered via a filter paper (paper no: 2727). This step was repeated two times.
b. 100 mL of methanol was added to the propolis taken into the flask and mixed. Afterward, the samples centrifuged at 4000 × g/1 min.
c. At the end of the process, the mixture was filtered with the help of filter paper. The liquid extracts obtained after the filtration process were stored at +4°C before the analyzes to be made. Organic substances are generally more soluble in organic solvents than in water. By utilizing these properties, organic substances dissolved in water are taken into the organic solvent. Solvents are changed for extraction.

The bioactive components obtained in extraction should be obtained without loss and degradation and without the need for additional purification. In liquid extraction, which is based on the principle of obtaining one or a part of the components of a solid substance using a suitable solvent, efficiency is affected by factors such as the solvent type and pH [20]. Changing the solvent after the first step is done in order to perform the appropriate fractionation for LCMS/MS and to collect compounds with higher efficiency.

### 2.2. Determination of the Phenolic Content of Propolis Samples

The phenolic content of all propolis samples was determined with LC MS/MS [21, 22].

The stock solution (1 mg/mL) of chemical substances (caffeic acid, caffeic acid phenethyl ester, 1,1-dimethyl allyl ester caffeic acid, benzyl ester caffeic acid, ferulic acid, salicylic acid, gentisic acid, catechin, chlorogenic acid, vanillic acid, and ethyl ferulate) were prepared using acetonitrile (ACN) and diluted in concentrations range of 1–10,000 ng/mL with water/acetonitrile (50/50:v/v) mixture containing 0.1% formic acid. Salicylic acid was diluted in concentrations range of 1–1000 pg/mL. The calibration curves were drawn for each chemical.

The quantitative analyses were performed by using the Waters-ACQUITY TM TQD tandem quadrupole UPLCMS/MS system, with ACQUITY TQ detector in the electrospray ionization (ESI) and multiple reaction monitoring (MRM) mode (Waters, Milford, MA) option. This UPLCMS/MS system was conducted by MassLynxTM 4.1 software.

After separation with a Waters Acquity UPLC BEH C18column (2.1 mm 9 50 mm, 1.7 lm, Waters, Milford, MA, USA) samples were run in mobile phase A (0.2% formic acid in water (v/v) and mobile phase B (0.1% formic acid in ACN)). The column temperature was 60°C and the autosampler temperature was 10°C. For MRM data collection, the capillary voltage was 3730 V and the source temperature was 150°C. The cone gas flow was 40 L/h, and the desolvation gas flow was 600 L/h [19].

### 2.3. Studies in Cell Culture

Human breast cancer cell line MCF-7 (ATCC HTB-22), human colorectal cancer cell line SW-620 (ATCC CCL-227), human prostate cancer cell lines DU-145 (ATCC HTB-81), and PC-3 (ATCC CRL-1435) were used in this study, and healthy fibroblast cell line WI-38 (ATCC CCL-75) was used as control. All cell lines were taken from the cell line stock of Ege University Faculty of Medicine, Department of Medical Biology. While previous studies have only focused on breast cancer, we aimed to screen a wider area by using prostate cancer types (DU-145 and PC-3) and an aggressive colon cancer cell line (SW-620) in addition to breast cancer cell line (MCF-7). The reason why we chose different cancer cell lines in this study is to determine the most effective treatment type of PPPR21A when propolis is applied in different cancer cell lines and to continue further studies more intensively in line with the results we obtained.

Cells were grown in 75 cm^2^ flasks in Roswell Park Memorial Institute (RPMI) 1640 medium (for SW-620 cells), Eagle's Minimum Essential Medium (EMEM, for DU-145, PC-3, and MCF-7 cells), or Dulbecco's Modified Eagle Medium/Nutrient Mixture F-12 (DMEM/F-12). The media were supplemented with 10% fetal bovine serum (FBS, Biological Industries, Cat. No: 04-127-1A), 1% (2 mM) L-Glutamine (Biological Industries, Cat. No: 03-020-1B), and 1% Penicillin-Streptomycin (Biological Industries, Cat. No: 03-031-1B). The cells were incubated at 37°C in a sterile cell culture incubator with 95% humidity and 5% CO_2_.

The replicated cells were regularly monitored for viability, proliferation, and infection using an inverted microscope. The trypan blue staining method was used to control cell viability. Trypan blue stain will be applied to check the viability and number of cells. For this purpose, 50 μL of dye is mixed with 50 μL of suspended cells and the viability and number of cells are examined with a “Neubauer” slide under a light microscope. Four areas consisting of 4 × 4 squares on the “Neubauer” slide are counted. Cells that contain the trypan blue dye and appear blue in color because the integrity of the cell membrane is disrupted are considered dead, and cells with intact cell membrane structure are considered alive because they do not contain the dye. The number of live cells in ml is calculated by taking the average of the totals of the counted live cells and multiplying by the dilution factor and 20.000. Cell viability will be calculated by proportioning the number of viable cells to the total number of cells as %.

#### 2.3.1. Cell Proliferation Assay–Real-Time Cell Analysis System (xCELLigence)

Cell proliferation assay was set up to determine the appropriate number of cells per mL of SW-620, DU-145, PC-3, MCF-7, and WI-38 cells for use in further molecular analysis. The real time cell analysis system (xCELLigence) (RTCA-SP) device and its software were used to determine cell viability.

With this method, changes in cell biology are followed in real time. The cell index (CI) parameter is used to express cell viability. The CI value is a quantitative measurement that varies with viable cell density. The CI value shows zero (0) when there are no cells in the medium. At the time of cell proliferation, the CI value increases from 0 and takes value depending on the cell density.

##### 2.3.1.1. Cytotoxicity Assays: xCELLigence

The xCELLigence system was used as described in the proliferation experiment to determine the cytotoxic effect of propolis samples in SW-620, DU-145, PC-3, MCF-7, and WI-38 cell lines.

Cells, determined by the optimal cell number proliferation assay, were seeded in 96-well plates in 200 μL of medium in replicates of 3. After incubation in an incubator for 24 h, propolis samples were given to the cells in 6 different concentrations (250, 300, 350, 400, 450, and 500 μg/mL) selected according to the data we obtained in our previous studies [23] and the literature, with a final volume of 200 μL of medium. After the propolis addition into cell culture, the device software made a measurement every 15 min for 72 h. Cells without active substance were used as the control group. Using the CI values obtained for each well, the % cell viability and % cytotoxicity values at 24, 48, and 72 h were calculated according to the following formula [24]:

(1) $$\begin{array}{c} \%\text{ Cytotoxicity}=100–\left[\frac{\text{average cell viability in active substance}-\text{treated wells}}{\text{average of cell viability in control wells }\left(\text{no active substance applied}\right)}\right]\times 100. \end{array}$$

Calculation of IC_50_ values was obtained by the RTCA Software Version 1.2.1.

#### 2.3.2. Determination of PPPR2A and Apoptosis in Cell Culture

The IC_50_ concentrations (298, 185.6, 250.7, 292.9, and 311.2 μg/mL) of propolis samples were applied to SW-620, DU-145, PC-3, MCF-7, and WI-38 cells at 80% occupancy in 75 cm^2^ flasks and the changes in Human PPP2R1A (REF: DZEDRB-T-86642) levels were determined using 96T BioSence Elisa Kit. In the ELISA method, 3 replicates will be performed using the standard specified in the kit as the standard. Ten μL of biotin will be added to all samples and then 50 μL of streptavidin will be added to all samples and standards except blank. It will then be incubated at 37° for 60 min. After this, wash 5 times and then add 50 μL of chromogen A and B and incubate for another 10 min. The reaction will then be stopped with 50 μL of stop reagent and the measurement will be performed spectrophotometrically (OD) at 450 nm.

APAF-1, caspase-3, caspase-8, caspase-9, cytochrome-c, and TRAIL levels were determined.

For all cell lines, cells were seeded in 6-well plates and the experimental group was treated with the IC_50_ concentration of propolis samples for 48 h, and the cells without substance were used as the control group.

### 2.4. Statistical Analysis

The IC_50_ value, defined as the inhibitory concentration of the substance causing a 50% reduction in cell proliferation, was determined by the xCELLigence device software. Cytotoxicity graphs for time and concentration dependent dose effect values were drawn with Graphpad v.8.0 program. Statistical differences between control and experimental groups were analyzed using one-way ANOVA in Graphpad v.8.0, followed by Dunnett's *t*-test, and *p* < 0.05 values were considered significant. The control group and experimental group values were analyzed by the Student *t*-test, and the significance value *p* < 0.05 was determined. Experiments were carried out in triplicate.

## 3. Results

### 3.1. Phenolic Content of Propolis Is Shown in Table 1

By the LC MS/MS method, 24 phenolic molecules were analyzed and it was found that caffeic acid, ferulic acid, apigenine, pelargonine, cyanidin, genistein, naringenin, and dmaecape (1,1-dimethyl allyl caffeic acid ester) were found in higher concentrations in the propolis extract than the others.

### 3.2. Cytotoxic Effect of Propolis on Different Cancer Cell Lines and Healthy Cell Line

**Figure 2:**
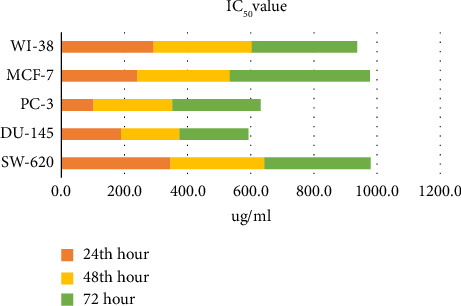
24, 48, and 72 h cytotoxic effects of the propolis sample on colon cancer (SW-620), healthy (WI-38), prostate cancer (DU-145), prostate (PC-3), and breast cancer (MCF-7) cell lines (*p* < 0.001).

The cytotoxic effect of the propolis sample on colon cancer (SW-620), prostate cancer (DU-145, PC-3), breast cancer (MCF-7), and healthy (WI-38) cell lines was determined in real time with the xCelligence RT-SP device. Accordingly, increasing dose-related % cytotoxicity values and IC_50_ values on SW-620, DU-145, PC-3, MCF-7, and WI-38 cell lines, for the 24^th^, 48^th^, and 72^nd^ h, as stated in Table 2 and Supporting Information Figures 1–9, the data are shown in Figure 2.

The IC_50_ values obtained for 24, 48, and 72 h in propolis, PC-3, and DU-145 human prostate cancer cell lines are smaller than the IC_50_ value obtained for WI-38 human healthy lung fibroblast cells. Therefore, propolis showed higher cytotoxic activity against PC-3 and DU-145 human prostate cancer cells for 24, 48, and 72 h than WI-38 human healthy lung fibroblast cells. The selectivity index (SI) is calculated as the ratio of the healthy cell IC_50_ value to the cancer cell IC_50_ value. High SI (especially between 1 and 10) is a very important feature for molecules that may be drug candidates. After 24 h and 48 h propolis application, SI decreases by approximately 1.5, 72 h after application. For our study, we accepted the time at which all cell lines have approximately the same SI and 48^th^ h IC_50_ propolis concentrations with a SI of approximately 1.5 were selected for all cell lines.

### 3.3. Effect of Propolis on PPP2R1A

SW-620, DU-145, PC-3, MCF-7, and WI-38 cells were incubated with propolis at 298, 185.6, 250.7, 292.9, and 311.2 μg/mL concentrations, respectively, according to the IC_50_ values determined in the preliminary studies, and their PPPR21A levels and apoptosis markers were determined. PP2R1A levels are presented in Table 3.

Before addition of propolis, PPP2R1A levels were lower in all cancer cell types than those in control cells (WI-38). No significant difference was observed in WI-38 cells after propolis addition.

Although there was a 1.17-fold increase in DU-145 cells after propolis addition, no statistically significant change was observed. In addition, our data showed that propolis treatment significantly increased PPP2R1A levels in SW-620 cells (1.86-fold), PC-3 cells (1.17-fold), and MCF-7 cells (1.2-fold) compared with cells without propolis addition.

### 3.4. Effect of Propolis on Apoptosis

**Figure 3:**
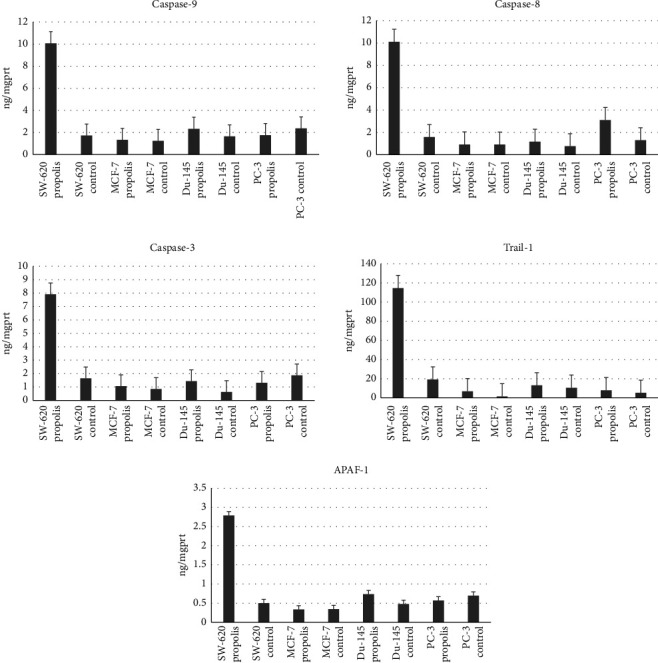
Apoptosis levels in cancer cell lines and healthy cell line of propolis samples (*p* < 0.05). SW-620 propolis, DU-145 propolis, PC − 3 propolis, and MCF-7 propolis: apoptosis marker levels in cancer cells obtained by application of propolis with IC_50_ dose determined. SW-620 control, DU-145 control, PC − 3 control, and MCF-7 control: apoptosis marker levels in cancer cell lines without propolis treatment.

Propolis treatment increased all apoptosis markers in SW-620 cells and DU-145 cells compared with those of no propolis added cells. (*p* < 0.05) (Figure 3).

There was an approximately 1.5-fold increase in caspase-8, caspase-3, and TRAIL level in PC-3 cells supplemented with propolis compared with cells without propolis (*p* < 0.05).

There was a 4.63-fold increase in the TRAIL level in MCF-7 cells supplemented with propolis compared with cells without added propolis (*p* < 0.05) (Figure 3).

When all apoptosis markers are evaluated, there is no significant difference in the healthy cell line before and after the application of propolis. However, when we compared the healthy cell lines to cancer cell lines, a statistically significant increase in caspase-3 (3.62-fold) and in TRAIL (4.38-fold) was observed in the SW-620 cell line after the application of propolis. In addition, in the PC-3 cell line, a 1.4-fold increase in caspase-8 was observed compared to the healthy cell line, which is also statistically significant (*p* < 0.05) (Figure 3).

## 4. Discussion

Our previous studies have shown the antitumor effects of propolis on cancer cell lines, especially on colon cancer cell lines [23, 25, 26]. This study, which was carried out with the idea that propolis suppresses cancer proliferation and drives cancer cells to apoptosis via PPPR21A in both colon cancer cell lines and other cancer cell lines, completes an effective gap in the elucidation of the mechanism of action of propolis. Recent studies have demonstrated a role for PP2A in human tumorigenesis, showing that normal PP2R1A activities may protect cells against transformation into cancer cells [27].

Turrini et al. [28] identified inactivating mutations in the PPP2R1A gene in lung and colon carcinomas, illustrating its ability to suppress tumor development through its role in cell cycle regulation and cellular growth control. In this study, we investigated the effect of propolis as a potential stimulant on PPP2R1A levels, targeting various cancer cell lines.

Studies on different histologic subtypes of ovarian and uterine neoplasms showed that mutation degree in PPP2R1A were associated with the grade and type of cancer [29]. Their study revealed that uterine (type II) high-grade serous carcinomas have had higher prevalence of PPP2R1A mutations than ovarian (type II) high-grade serous carcinomas [30]. In our study, the lowest PP2R1A levels were observed in MCF-7 cells but it is not comparable with the human studies. We used the SW-620 cell line as an in vitro model to investigate important cellular functions, especially metastasis. Therefore, SW-620 cells showed the most obvious variation in our investigation, which may be related to the pathway mentioned above.

As is well known, PP2A functions as a tumor suppressor and is crucial in maintaining cellular signal homeostasis by phosphorylating a variety of signaling proteins, and protection of PP2R1A levels is one of the targets of cancer treatment.

In this study, we observed that PPP2R1A levels were lower in all cancer types compared to healthy cell lines, which is consistent with the literature suggesting that PPP2R1A levels are associated with the clinical stages and prognosis of various cancer types [31]. Notably, we demonstrated for the first time that propolis treatment resulted in a 1.86-fold increase in PPP2R1A levels in the colon cancer cell line SW-620 compared with untreated cells. Our data demonstrated that propolis treatment significantly increased PPP2R1A levels in SW-620 cells (1.86-fold), PC-3 cells (1.17-fold), and MCF-7 cells (1.2-fold) compared with untreated cells (Table 3).

The authors have proposed different mechanisms for the protective effect of overexpressed PP2R1A on cancer invasion and proliferation. Sui and colleagues suggested that PP2A is important for telomere maintenance. They showed that the binding of PP2A to its scaffold subunit (PP2R1A) promotes the hnRNPA1 (heterogeneous nuclear ribonucleoprotein A1)-to-POT1 (protection of telomeres 1) transition by dephosphorylating hnRNPA-1 in late M phase, thereby protecting telomeric single-stranded DNA from Rad3-associated damage [31].

Hou et al. [32] proposed another mechanism, observing that the WNK1-OSR1-PPP2R1A axis was crucial for enhancing tumor-induced angiogenesis in both endothelial and hepatoma cells. In their study, overexpression of PPP2R1A counteracted the cell migration induced by increased WNK1 levels in HepG2 hepatoma cells. They suggested that PPP2R1A acted as a downstream effector in hepatoma, mitigating the enhanced cell migration driven by WNK1, a key protein kinase involved in tumor-induced angiogenesis. In addition, Chi et al. [33] provided supporting evidence by demonstrating that the reduction or deletion of WNK1 led to increased cytoplasmic retention of Forkheadbox protein O (FOXO1), decreased PP2A activity, and subsequently, AKT hypersignaling, which promotes epithelial proliferation. Research on various histologic subtypes of ovarian and uterine neoplasms has demonstrated that the mutation degree in PPP2R1A correlates with the grade and type of cancer [34, 35]. This study found that uterine (type II) high-grade serous carcinomas exhibited a higher prevalence of PPP2R1A mutations compared with ovarian (type II) high-grade serous carcinomas [29]. In our study, the lowest PP2R1A levels were observed in MCF-7 cells. The SW-620 cell line, used as an in vitro model to investigate essential cellular functions for metastasis, showed the most significant variation in our investigation, which may be linked to the aforementioned pathway.

Many studies have been conducted to show the induced effect of natural products on the PP2A levels until now. Different studies have reported that PP2A activity is frequently inactivated in various tumors and causes abnormal activation of signaling pathways associated with tumor growth [36]. The AKT/mTOR signaling axis is frequently hyperactivated in many types of cancer and is involved in key processes such as proliferation, metabolic adaptation, autophagy, and apoptosis. Supporting this, the study aimed to show that Cory, a natural product derived from the Chinese herbal medicine *Uncaria rhynchophylla*, inhibits the proliferation of NSCLC cells in vivo and in vitro and demonstrated that Cory increases PP2A activity and suppresses the AKT/GSK3*β* signaling pathway to induce cellular apoptosis in NSCLC, while inhibiting the AKT/mTOR signaling pathway that triggers autophagy. In particular, the activation of PP2A inhibited AKT, indicating that it plays a crucial role in the antitumor effects of Cory [36]. In another study using naturally occurring protein kinase C (PKC) activators such as phorbol esters, teleosidins, and aplysiatoxins, in vitro analysis using A549, one of the aplog-sensitive cell lines, revealed that PKC*α* induced PP2A-mediated attenuation of the Akt/S6 signaling axis [37]. In a study using (+)-cyanidan-3-ol (CD-3) as a selective compound to inhibit the growth of squamous cell skin cancer (SCSC) cell lines, we found that CD-3 inhibitory effects on SCSC growth were mediated through cell cycle arrest and caspase-dependent induction of apoptosis and in mechanistic studies. We showed that CD-3 activates PP2A by inhibiting CIP2A and produces tumor growth inhibitory effects by promoting dephosphorylation of oncogenic AKT/mTOR signaling proteins in SCSC cells and xenograft tumors in a PP2A-dependent manner. Furthermore, the combination of CD-3 and mTOR inhibitors (mTORi) synergistically reduced oncogenic phenotypes [38].

These studies promised that the induction of PP2A activation with different mechanisms in terms of inhibiting AKT/mTOR signaling, or Erk1/2 mediated adhesion of cancer cells, might inhibit the growth of cancer cells as well as invasion of cancer [39]. Teiten et al. observed that curcumin showed anticancer effect by modulating several proteins involved in crucial cellular processes, including protein folding (heat shock protein PPP2R1A), RNA splicing (RBM17 and DDX39), cell death (HMGB1 and NPM1), and androgen receptor signaling (NPM1 and FKBP4/FKBP52) in androgen-dependent (22Rv1) and androgen-independent (PC-3) human prostate cancer cell lines. Teiten and colleagues demonstrated that curcumin may influence the expression of microRNAs such as miR-141, miR-152, and miR-183. Collectively, these findings support the potential of curcumin as a chemopreventive agent due to its ability to modulate the expression of proteins implicated in prostate carcinogenesis [40]. In this study, we showed that propolis induced PP2R1A levels up to 1.17-fold in PC-3 and 1.17-fold in DU-145 prostate cancer cell lines compared with untreated cells (*p* < 0.05). Propolis treatment also led to an increase in the levels of caspase-3, caspase-8, and TRAIL in these cells (Figure 3).

In our study, we observed a 1.2-fold increase in PPP2R1A levels in the MCF-7 breast cancer cell line treated with propolis compared with untreated cells (*p* < 0.05). Similarly to our data, another study [41] showed an increase in PP2A levels in MCF-7 and MDA-MB-231 cells treated with eribulin. They found that eribulin-induced phosphorylation of stathmin in MCF-7 and MDA-MB-231 cells was attenuated by a protein kinase A inhibitor (H89) and a Ca2+/calmodulin-dependent kinase II inhibitor (KN62). In addition, phosphorylated stathmin expression was reduced by the protein phosphatase PP2A activator FTY720 but increased by the PP2A inhibitor okadaic acid. Notably, while eribulin did not directly affect the phosphatase activity of recombinant PP2A, the expression of PP2A subunits was decreased in cells treated with eribulin. Furthermore, the antiproliferative effect of eribulin was stronger in stathmin-expressing cells. These findings suggest that stathmin dynamics are closely related to the antiproliferative effects of eribulin and that stathmin may serve as a potential biomarker to predict the therapeutic effects of eribulin in breast cancer patients [42].

In this study, we determined the apoptotic markers after propolis supplementation to demonstrate a possible relationship between PP2R1A activation and apoptosis. We observed an increase in apoptotic markers in all cell lines treated with propolis compared with untreated cell lines. Propolis treatment significantly increased the levels of apoptotic markers, particularly caspase-8 and TRAIL, in SW-620, DU-145, PC-3, and MCF-7 cell lines, suggesting induction of the extrinsic pathway of apoptosis as described in the literature [9]. Interestingly, the most pronounced effect of propolis on PP2R1A levels and apoptotic markers was observed in SW-620 cell lines with high metastatic capacity, indicating that propolis could inhibit the invasion of cancer cells by inducing the external pathway of apoptosis mediated by PP2R1A activation. In accordance with these findings, Li et al. observed that the overexpression of PPP2R2A promoted cell apoptosis and the expression of BAX, CAS-3, CAS-9, and the BAX/BCL-2 ratio in endometrial cells [15]. It has been found that the downregulation of PPP2R2A, along with the downregulation of CDK2, CDK4, CCND1, and CCNE1 and upregulation of P21, significantly reduced cell proliferation by preventing cell cycle transition from G0/G1 to S phase. Furthermore, G2/M phase arrest was primarily induced by a low dose of 7HF through the upregulation of Bub3, cyclin B1, phosphorylated Cdk1 (Tyr 15), and p53-independent p21 expression in TNBC cells. Conversely, increased expression of the PP2A-A subunit may have influenced the suppression of several cell survival proteins, including FoxO3a, p-Akt (Ser 473), and *β*-catenin. The apoptotic effect of 7HF on treated cells was mediated by upregulating Bax and active cleaved caspase-7-9 expression and downregulating Bcl-2 and full-length caspase-7-9 expression, through both intrinsic and extrinsic pathways. Proteomic analysis, in particular, revealed upregulation of significant protein clusters associated with apoptosis, G2/M-phase transition, and G1/S-phase arrest [43]. By suppressing pathways including ERK1/2, Akt, NF-κB, Jun-N terminal kinase, and Wtn2 and FAK as well as MAPK and PI3K/AKT signaling pathways, propolis can stop angiogenesis while also slowing the spread of cancer metastatically. Furthermore, propolis or its constituent parts have regulatory actions on CDK2/4/6, cyclin D, and their inhibitors. In addition, cell cycle arrest in G2/M or G0/G1 may be caused by propolis-induced upregulation of p21 and p27. Propolis has a wide antiapoptotic impact that is achieved by downregulating the ERK1/2 signaling pathway and upregulating TRAIL, Bax, and p53. Propolis is a natural substance that may be used as an adjuvant therapy to help lessen the negative effects of radiation and chemotherapy. This is because there is increasing evidence about the anticancer properties of propolis and its active ingredients [44].

The ability of various forms of propolis to induce apoptosis has been studied in the past [45]. It was discovered that ethanolic extract of propolis (EEP) may downregulate the extracellular signal-regulated kinases (ERK1/2) signaling pathway and trigger a number of cascades, such as the Bcl-2 associated X protein (Bax) and p53 pathway, the TRAIL pathway, and others [46]. According to Jiang et al., at concentrations as low as 25–50 μg/mL, Chinese propolis induced apoptosis by upregulating Bid, downregulating Bcl-2, and activating p53 and Bax. Cytochrome-c was produced during this process, activating poly (ADP-ribose) polymerase (PARP) and cleaved caspase-3/8/9 [47]. Frión-Herrera et al. found that extracts of Brazilian green propolis (BP) and Cuban red propolis (CP) show antiproliferative effects at concentrations between 50 and 100 μg/mL by inducing apoptosis and producing reactive oxygen species (ROS) by downregulating BCL-XL, PUMA, and Bcl-2 and upregulating caspase-3, TP53, Bax, and p21 [48].

Regardless of the kind of cancer, propolis has been shown to trigger apoptosis in a variety of cancer cell lines by activating components in both intrinsic and extrinsic pathways [42]. It has been demonstrated that propolis can cause apoptosis by activating caspases-3, -8, and -9, decreasing TRAIL resistance, and increasing the release of cytochrome-c [49]. For the first time, we assessed the correlation between the rise in PPPR21A and the rise in molecules implicated in the apoptotic pathway in several cancer cells in order to explore the potential protective impact of propolis samples. The levels of caspases-3, -8, and -9 and TRAIL were favorably linked with phenolic compounds (ferulic acid, caffeic acid, kaempherol, myricetin, transcinnamic acid, ellagic acid, CAPE, DMEA caffeic acid, and quercetin) in propolis samples [50]. In this study, propolis significantly increased caspase-8, cytochrome-c, and TRAIL levels. They also showed that propolis significantly increased APAF-1 levels. Research indicates that kaempherol, quercetine, caffeic acid, transcinnamic acid, ellagic acid, CAPE, DMEA, and miricetine are also linked to the APAF-1 level [23]. To the best of our knowledge, kaempherol, miricetine, transcinnamic acid, ellagic acid, CAPE, DMEA, caffeic acid, and quercetine are all strongly associated with this propolis action [51]. Because propolis reverses the major molecule levels in both the intrinsic and extrinsic pathways of apoptosis, it was found to be effective against cancer cell types in this investigation. This study was similar to the abovementioned studies. Propolis treatment increased all apoptosis markers in SW-620 cells and DU-145 cells compared with those of no propolis added cells (*p* < 0.05). There was an approximately 1.5-fold increase in caspase-8, caspase-3, and TRAIL level in PC-3 cells supplemented with propolis compared with cells without propolis (*p* < 0.05). There was a 4.63-fold increase in the TRAIL level in MCF-7 cells supplemented with propolis compared with cells without added propolis (*p* < 0.05).

## 5. Conclusion

It has been shown that common genetic alterations in PPP2R1A (which encodes the inhibition of PP2A activity), affecting up to 40%, promote malignant transformation in the tumor-suppressing heterotrimeric PP2A A*α* subtype. The endogenous inhibitory mechanisms of PP2A have been associated with malignant progression and prognosis in various cancers. Consistent with these studies, low levels of PPP2R1A have been found in cancer cells. According to our results, after the addition of propolis, a 1.17-fold increase was observed in DU-145 cells, but no statistically significant change was detected. In addition, our data showed that propolis treatment significantly increased PPP2R1A levels in SW-620 cells (by 1.86 times), in PC-3 cells (by 1.17 times), and in MCF-7 cells (by 1.2 times) compared with cells without propolis. It was observed that propolis treatment did not change the levels of PPP2R1A in healthy cells but increased them in cancer cells. In our study investigating the relationship between propolis and cancer cell lines' PPP2R1A and apoptosis, the treatment with propolis supports our findings positively, as it increases all apoptosis markers in SW-620 and DU-145 cells compared with cells without propolis. In this study, the inducible effect of propolis on PP2R1A levels has been demonstrated for the first time as a new target for cancer treatment. This situation suggests that propolis shows promise in cancer treatment and demonstrates its ability to increase PPP2R1A levels, thereby affecting the target molecule in cancer therapy.

## Figures and Tables

**Table 1:** Phenolic content of propolis samples.

Propolis	Caffeic acid	Ferulic acid	Gentisic acid	Apigenin	Pelargonin	Cyanidin
ng/mL	439.02	453.106	9895	382.28	360.63	890.15
Propolis	Trans-cinnamic acid	P-coumaric acid	Vanilic acid	Ethyl ferulate	DMAECAPE	Naringenin
ng/mL	92.58	36.49	52.02	3.00	401.57	341.07
Propolis	Kaempferol	Rutin	Epichatechin	Quercetin	Ellagic acid	Delphinidin
ng/mL	7987	0.844	1.65	3.56	1.17	13.97
Propolis	Salicylic acid	Prochatechoic acid	Genistein	Chlorogenic acid	Amentoflavone	Maleic acid
ng/mL	0.30	3361	294.04	1710	1113	2.12

*Note:* The results are equivalent to nanograms of phenolic molecules in 1 mg of propolis.

**Table 2:** IC_50_ values (μg/mL).

Cell line	24^th^ hour	SI	48^th^ hour	SI	72 h	SI
SW-620	345.00	0.85	298.00	1.04	337.00	0.99
DU-145	189.40	1.54	185.60	1.68	217.20	1.53
PC-3	101.20	2.89	250.70	1.24	279.40	1.19
MCF-7	240.80	1.22	292.90	1.06	443.20	0.75
WI-38	292.60		311.20		333.30	

*Note:* Selectivity index (SI) was calculated as the ratio of the IC_50_ value calculated for WI-38 healthy cells to the IC_50_ value calculated for cancer cell lines.

**Table 3:** PPP2R1A levels in cancer cell lines and healthy cell lines.

Cell line	PPP2R1A	PPP2R1A
	(pg/mg prt) control	(pg/mg prt) propolis
WI-38	4124.42	4152.55
SW-620	2590.91	4822.45⁣^∗^
DU-145	1880.85	2203.29
PC-3	1517.23	1775.49⁣^∗^
MCF-7	1350.26	1620.87⁣^∗^

*Note:* PPP2R1A levels in cancer cell lines and healthy cell lines were measured to evaluate the effect of propolis. PPP2R1A levels in cancer cell lines and healthy cell lines were compared.

Abbreviation: mg protein, milligram protein.

⁣^∗^*p* < 0.05, comparisons were made against their respective controls.

## Data Availability

All data and materials are available in Ege University Hospital Medical Biochemistry Department (Inflammation Studies), Ege University Hospital Medical Biology Department (Cell culture studies), and Ege University Food Engineering Department (propolis and extraction processes).

## Supporting Information

The cytotoxic effect of the propolis sample on colon cancer (SW-620), prostate cancer (DU-145, PC-3), breast cancer (MCF-7), and healthy (WI-38) cell lines was determined in real time with the xCelligence RT-SP device. Accordingly, increasing dose-related % cytotoxicity values and IC50 values on SW-620, DU-145, PC-3, MCF-7, and WI-38 cell lines for the 24th, 48th, and 72nd hours, as stated in the following, the data are shown in Figures 1–9.
