# Supplementary material for: Effect of Propolis on PPP2R1A and Apoptosis in Cancer Cells
Source: Biochem Res Int. 2025 Jan 15;2025:5538068. doi: 10.1155/bri/5538068 (PMC11756940; doi:10.1155/bri/5538068)
Supplement: Supporting Information — Additional supporting information can be found online in the Supporting Information section. [file 5538068.f1.docx]

**Cytotoxic Effect of Propolis on Different Cancer Cell Lines and Healthy Cell Line:**

The cytotoxic effect of propolis sample on colon cancer (SW-620), prostate cancer (DU-145, PC-3), breast cancer (MCF-7) and healthy (WI-38) cell lines was determined in real time with the xCelligence RT-SP device. Accordingly, increasing dose-related % cytotoxicity values and IC50 values on SW-620, DU-145, PC-3, MCF-7 and WI-38 cell lines; For the 24th, 48th and 72nd hours, as stated below, these data are shown in the Figure 1-9.


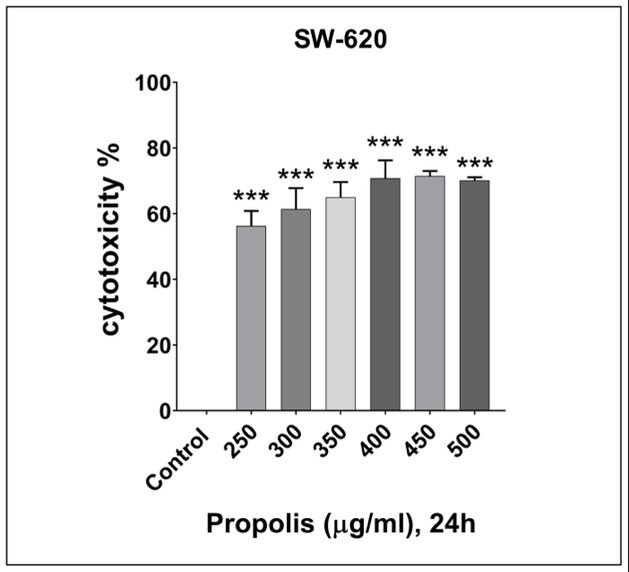

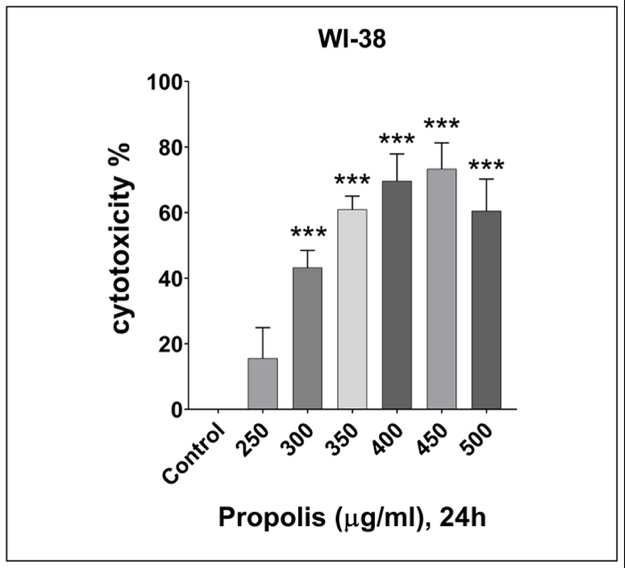


**Figure 1.** 24 hour cytotoxic effect of propolis sample on colon cancer (SW-620) and healthy (WI-38) cell lines (***p<0,001).


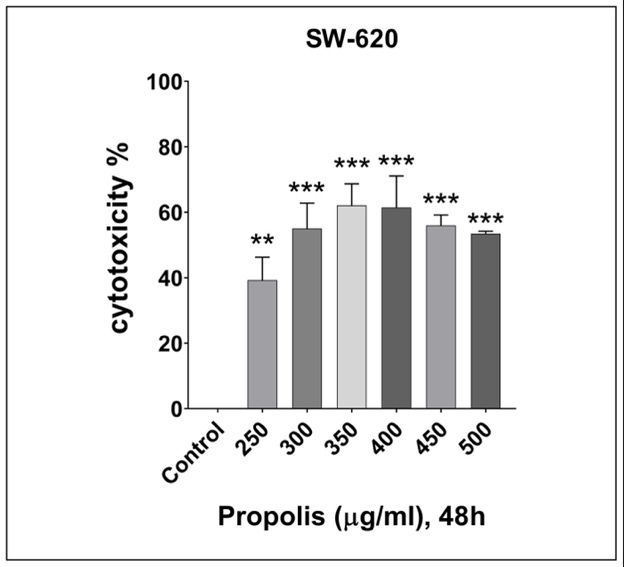

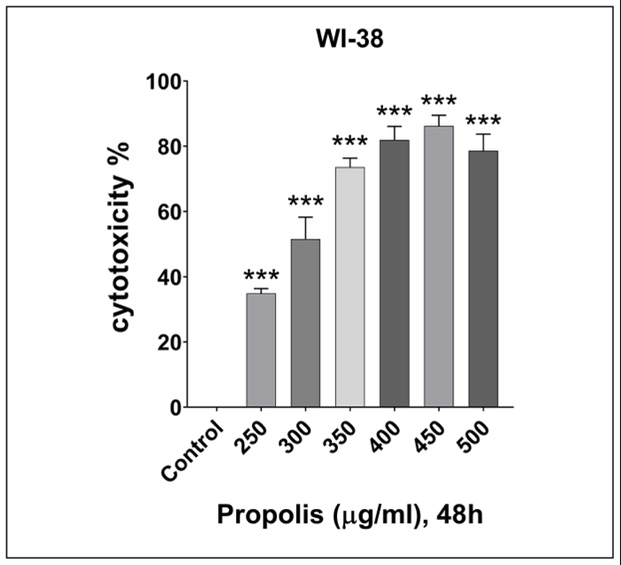


**Figure 2.** 48 hour cytotoxic effect of propolis sample on colon cancer (SW-620) and healthy (WI-38) cell lines (**p<0,01; ***p<0,001).


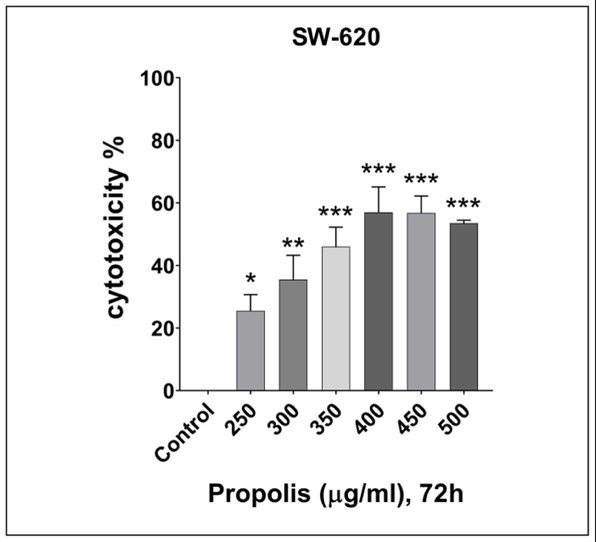

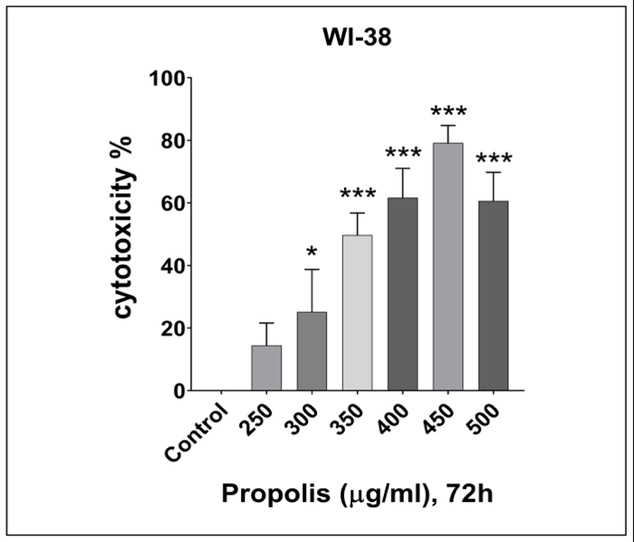


**Figure 3.** 72 hour cytotoxic effect of propolis sample on colon cancer (SW-620) and healthy (WI-38) cell lines (*p<0,05; **p<0,01; ***p<0,001).

IC50 values of the SW-620 cell line of the propolis sample:

It was found to be : **24th hour:** 345 µg/ml, **48th hour:** 298 µg/ml, **72th hour:** 337 µg/ml.

IC50 values of the WI-38 cell line of the propolis sample:

It was found to be: **24th hour:** 292,6 µg/ml, **48th hour:** 311,2µg/ml,**72th hour:** 333,3 µg/ml.


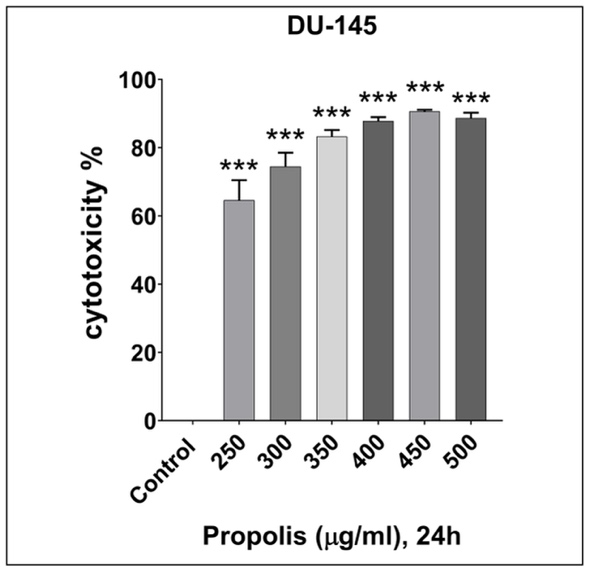

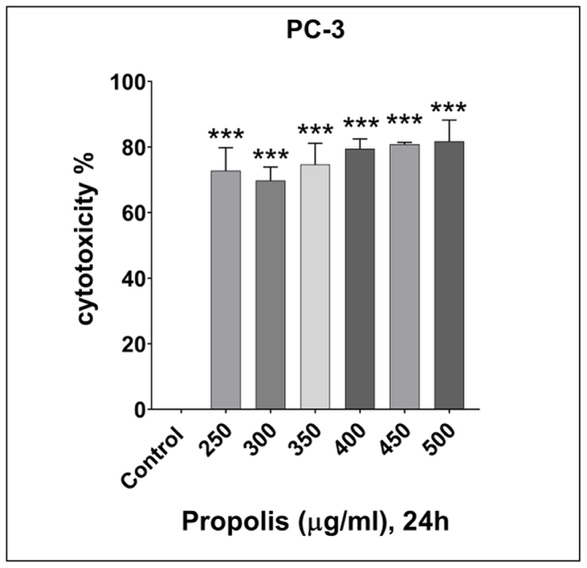


**Figure 4.** 24 hour cytotoxic effect of propolis sample on prostate cancer (DU-145) and prostate (PC-3) cell lines (***p<0,001).


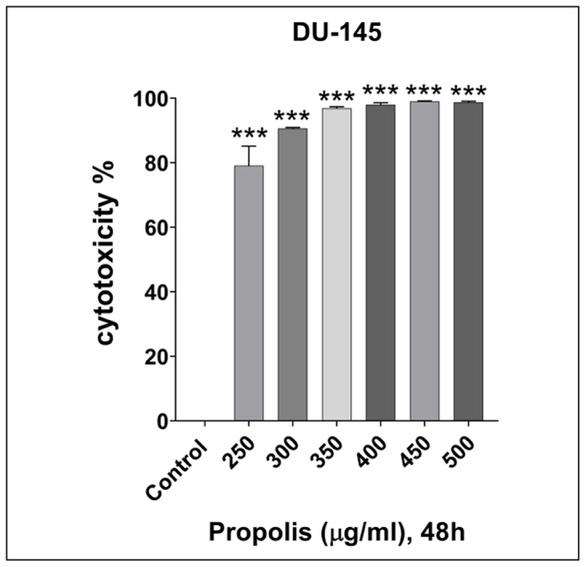

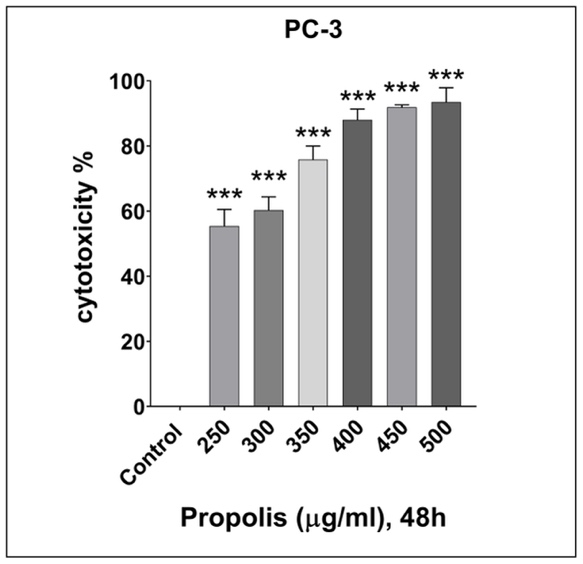


**Figure 5.** 48 hour cytotoxic effect of propolis sample on prostate cancer (DU-145) and prostate (PC-3) cell lines (***p<0,001).


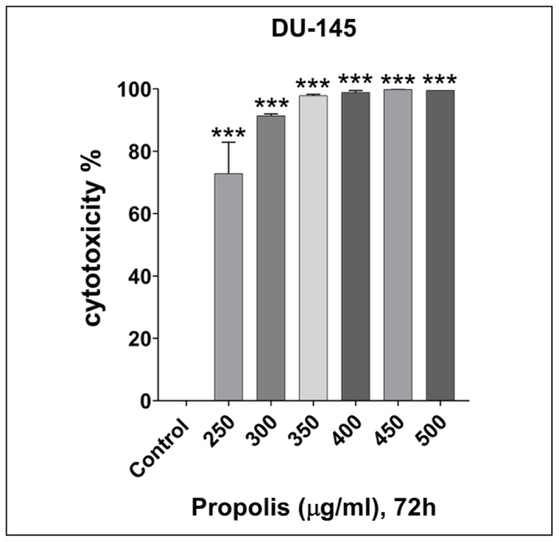

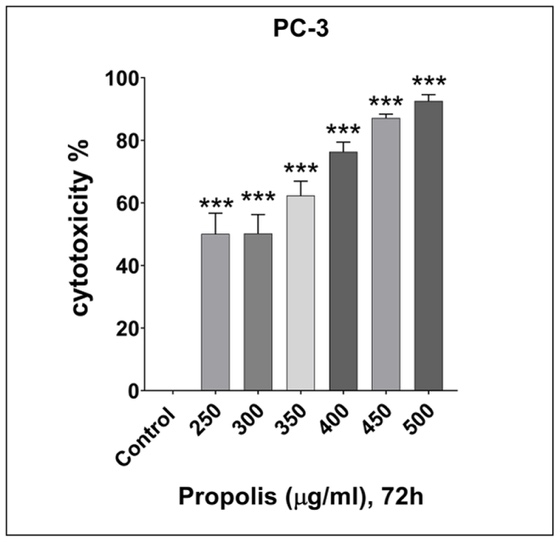


**Figure 6.** 72 hour cytotoxic effect of propolis sample on prostate cancer (DU-145) and prostate (PC-3) cell lines (***p<0,001).

IC50 values of the DU-145 cell line of the propolis sample:

It was found to be **24th hour:** 189,4 µg/ml, **48th hour:** 185,6 µg/ml, **72th hour:** 217,2 µg/ml.

IC50 values of the PC-3 cell line of the propolis sample:

It was found to be **24th hour:** 101,2 µg/ml, **48th hour:** 250,7 µg/ml, **72th hour:** 279,4 µg/ml.


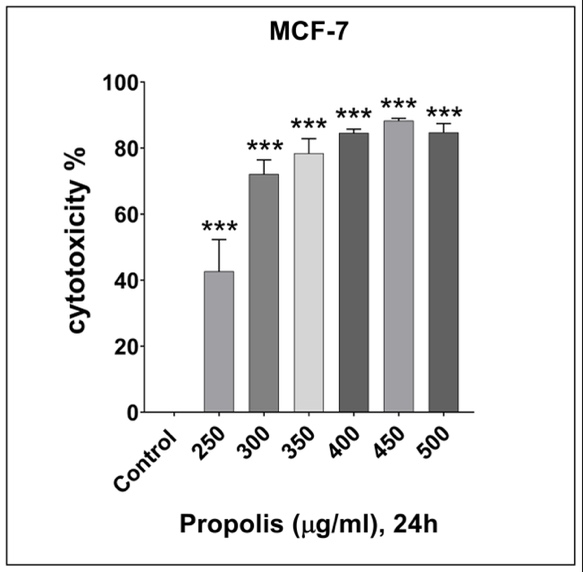


**Figure 7.** 24 hour cytotoxic effect of propolis sample on breast cancer (MCF-7) cell lines (***p<0,001).


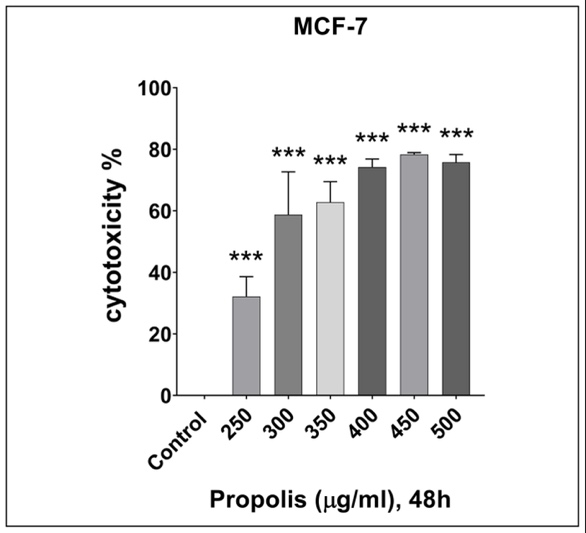


**Figure 8.** 48 hour cytotoxic effect of propolis sample on breast cancer (MCF-7) cell lines (***p<0,001).


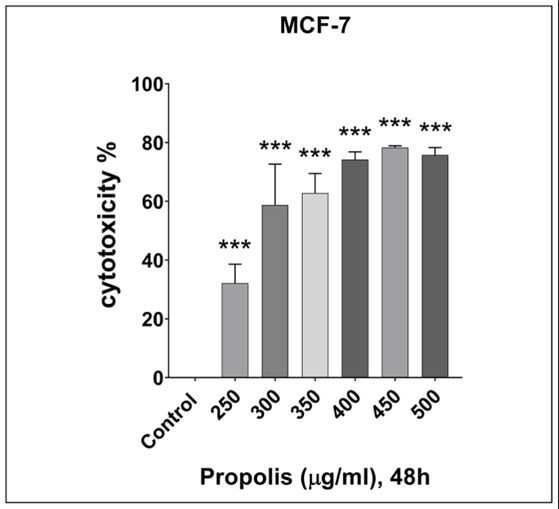


**Figure 9.** 72 hour cytotoxic effect of propolis sample on breast cancer (MCF-7) cell lines (***p<0,001).

IC50 values of the MCF-7 cell line of the propolis sample:

It was found to be **24th hour:** 240,8 µg/ml, **48th hour:** 292,9 µg/ml, **72th hour:** 443,2 µg/ml.
